# Social and organizational factors affecting implementation of evidence-informed practice in a public health department in Ontario: a network modelling approach

**DOI:** 10.1186/1748-5908-9-29

**Published:** 2014-02-24

**Authors:** Reza Yousefi-Nooraie, Maureen Dobbins, Alexandra Marin

**Affiliations:** 1 Health Research Methodology program, Faculty of Health Sciences, McMaster University, Hamilton, Canada; 2 School of Nursing and Department of Clinical Epidemiology and Biostatistics, McMaster University, Hamilton, Canada; 3 Department of Sociology, University of Toronto, Toronto, Canada

**Keywords:** Evidence-informed decision-making, Social network analysis, Information-seeking, Exponential random graph models

## Abstract

**Objective:**

The objective of this study is to develop a statistical model to assess factors associated with information seeking in a Canadian public health department.

**Methods:**

Managers and professional consultants of a public health department serving a large urban population named whom they turned to for help, whom they considered experts in evidence-informed practice, and whom they considered friends. Multilevel regression analysis and exponential random graph modeling were used to predict the formation of information seeking and expertise-recognition connections by personal characteristics of the seeker and source, and the structural attributes of the social networks.

**Results:**

The respondents were more likely to recognize the members of the supervisory/administrative division as experts. The extent to which an individual implemented evidence-based practice (EBP) principles in daily practice was a significant predictor of both being an information source and being recognized as expert by peers. Friendship was a significant predictor of both information seeking and expertise-recognition connections.

**Conclusion:**

The analysis showed a communication network segregated by organizational divisions. Managers were identified frequently as information sources, even though this is not a part of their formal role. Self-perceived implementation of EBP in practice was a significant predictor of being an information source or an expert, implying a positive atmosphere towards implementation of evidence-informed decision making in this public health organization. Results also implied that the perception of accessibility and trust were significant predictors of expertise recognition.

## Introduction

Evidence-informed decision making (EIDM) has been proposed and advocated as a ‘a complex, multi-disciplinary process that occurs within dynamic and ever-changing communities’, to fill the widely-recognized gap in the translation of research evidence into practice [1]. Structure, context, and system are important moderators of the knowledge translation (KT) process [2].

Several studies have shown that planned KT interventions which are informed by and tailored to the potential barriers and facilitators are more effective in sustainable use of research [3]. Many barriers work at levels beyond individual practitioners, including teams and social environments, organizations, or broader inter-organizational environments [4]. Interventions may not reach the target population; or the target population may not adopt them, because they are ‘imposed from outside’, or due to ‘organizational instability’ and ‘limited organizational support’ [5]. Consequently, studying the characteristics of the target organization, social norms, and preparedness for organizational change is important in developing tailored interventions to implement EIDM in health organizations.

In public health organizations, managers have the responsibility of managing and controlling programs and services. Managers also have financial and operational responsibility for programs. But apart from the administrative and supervisory roles, their functions and impacts in the process of EIDM has not been adequately investigated.

Social network analysis (SNA) is a perspective for studying the social processes by examining the interactions and links between people and communities. One important application of SNA is addressing the patterns of knowledge sharing and diffusion of innovations in organizations. In addition to the descriptive measures and graphical presentation of social networks, stochastic models, which can assess the role of variability due to chance, can explain complex social processes. Stochastic models can disentangle the effect of various individual, dyadic, and more complex variables on the formation of social behavior [6].

SNA can untangle several social and relational aspects of the KT process. Network theories of social capital [7] and social influence [8] can explain the role of social networks as opportunities and constraints which facilitate or impede the implementation of KT interventions. Social networks can be studied as components of KT interventions (*e.g.*, the role of local opinion leaders in promotion of KT interventions [9]), or as the process by which the knowledge is diffused through healthcare settings [10]. And ultimately, network analysis can help us understand how implementation of KT intervention may reshape and change social networks as the outcome of implementation.

One important perspective to study the social and relational aspects of KT is analyzing the effect of social networks as contextual factors influencing the implementation of KT interventions. Investigating the pattern of information seeking in making evidence-informed decisions in public health organizations provides insights into the subtle network of interpersonal relationships and social and contextual factors that influence behaviors. This study aims to model how managers and professional consultants seek information to inform evidence-informed decisions in a public health department in Canada.

### Theoretical framework

#### Information exchange in public health organizations

Information-seeking networks represent the pattern of connections, by which people share information and ideas and seek guidance and information [11]. Health practitioners generally turn to peers as sources of information [12], and are influenced by opinion leaders in adopting new innovations [13].

Choice of information source is based on various competing and interlacing mechanisms, and may differ by the nature of the problem, the connectivity of the individual, the structure of the organization, and shared norms and values. Borgatti and Cross developed a model for information seeking in organizations [14], which is based primarily on two social theories explaining the distribution of knowledge in organizations and the behavioral tendencies of network members: transactive memory [15] and social exchange theory [16]. Their model suggests that the likelihood of seeking information from a particular person in organizations is a function of: the extent to which an information seeker recognizes and values another person’s knowledge and skill in certain areas; accessibility of that person; and the potential cost of information seeking [14].

#### Knowing where knowledge is stored

In addition to personal memory, people use many external memory resources to improve information retrieval. Peers are one of the most important external memory devices. Through social interactions, the specialized individual memory devices act as a collective and transactive memory system. Group members can retrieve the required piece of knowledge from the transactive system if they know how to refer to that piece of information and who possesses the needed knowledge [15]. Consequently, knowing where the required information is stored in the organization is the first important factor that determines the choice of informant.

In public health organizations, some staff are trained professionals who can help practitioners find, appraise, and apply evidence to inform their practice. In different public health organizations, staff with various job titles may take the professional consultant role, including epidemiologists, project specialists, and consultants. According to their formal job definition, we expected that, these professional consultants are significantly important sources of information for managers, and are more likely to be recognized as experts in EIDM:

H1: Professional consultants are more likely to be sources of information and be recognized as experts than managers.

In addition, in some Canadian public health departments, there is an organizational division responsible for the management of public health programs and services, and supervising the department’s management team and staff to ensure the department is meeting the public health needs of the community. Consequently, we expect that the staff of the supervisory/administrative division are more likely to be consulted than the staff of other divisions, because other staff refer frequently to them through the process of making organization-wide decisions. Consequently, we proposed that:

H2: Staff of the supervisory/administrative division are more likely to be sources of information than others in the health department.

#### Recognizing the expertise of the source

Apart from the formal organizational roles and positions, it is also important that the seeker of knowledge positively evaluates the capabilities of the knowledge source in the required area. Knowing and valuing someone’s knowledge and expertise is an important predictor of information seeking [14]. This personal judgment may be affected by several factors, one of which could be the professional behavior of potential information sources. Studies on the social influence of local opinion leaders have shown that their impact is mainly through non-verbal and informal routes [17]. In ambiguous situations individuals compare themselves with socially powerful individuals, in order to reduce mental conflicts [18]. So the presentations of personal behavior may influence the evaluation of peers about one’s expertise. Because information seeking is about finding, evaluating, and applying research evidence to practice, we expect that the staff who implement the principles of evidence-informed practice are more likely to be chosen as information sources than others. Therefore, we hypothesized that:

H3: Staff who have higher evidence-based practice (EBP) implementation scores are more likely to be recognized as information sources and experts.

#### Accessibility and ease

More recent studies on transactive memory systems have shown that knowing where in the network the information is stored is not enough for successful retrieval of information [19]. Information seeking is constrained by the availability of a potential information source. Physical proximity, time-constraints, and the likelihood of engaging the informant to answer the seeker’s questions may affect the accessibility of the information sources [14,19]. We hypothesize that staff prefer to limit their information sources to a small number of people, and also tend to limit themselves within the boundary of their organizational division. These hypotheses were supported by other studies in healthcare organizations [20]. We hypothesized that:

H4: Staff in the same division were more likely choose each other as information source or recognize each other as experts than staff from different divisions.

#### Cost

The cost of getting information affects the choice of information source. According to social exchange theory, information exchange in organizations can be explained by economic principles, through which receiving information from an informant obliges the seeker to pay back in some way [16]. People recognize the informant’s superiority (in terms of knowledge and expertise) in exchange for the information they receive. People usually need to seek advice from experts. However, many people may be hesitant to play the status recognition game by deferring to prestigious figures, because it highlights their own lower status and lack of knowledge and exposes them to the judgment of superiors. They look for alternative connections for information, in order to reduce the likelihood of such negative effects. People may rely on peers with whom they have pre-existing informal connections, such as friendship, working in the same place, having the same rank in the organization, and sharing the same values, in order to minimize the cost of advice [21]. These types of homophilous connections may lead to higher reciprocity and lower polarization of advice networks, because people with similar problems understand each other’s concerns more easily [22]. These informal routes of connection are sometimes even more important than formal and professional connections in changing people’s behavior. For example, Zheng *et al.* found that in adopting electronic health records, physicians are more influenced by their friends than their professional peers [23]. Among various dimensions of homophily, similarity in gender, level of seniority, and formal position in the organization are consistently reported in various contexts [14]. This tendency to be connected to similar peers may not apply to the recognition of experts, because people may recognize certain peers as expert, even when they are not connected directly. We hypothesize that:

H5: Managers are more likely to seek information from other managers, than from professional consultants.

In addition, the existence of affective ties among people may interact with the information-seeking behavior. On the one hand, there is a close association between friendship and homophily. According to Granovetter, people who are closer to each other are more likely to have similar attributes and values [24]. On the other hand, the existence of positive affective ties makes the seeker more comfortable revealing his/her lack of knowledge, and makes the source more flexible and willing to share [25]:

H6: Staff are more likely to seek information from their friends.

H7: Staff are more likely to seek information from peers with more similar EBP implementation scores than from the peers with less similar scores.

The tendency of individuals to be connected to more similar and accessible, peers is also rooted in their psychological desire for reciprocation from others [26]. People prefer to be connected to peers from whom they can demand more attention and devotion in return, as opposed to high status staff who do not have enough time/motivation to return the attention of the staff turning to them seeking advice. This norm of reciprocity is more likely to form in dense and closed networks [27]. In these networks when actor A does a favor for actor B, actor B will feel an obligation to return the favor as a moral obligation [28]. Because the public health organization is a small community including smaller clusters, which are shaped by organizational divisions, we expect to observe the tendency towards reciprocity in information-seeking network. We do not necessarily expect to see a tendency towards reciprocation in the expertise-recognition network. Because it is more about an attitude, which its reciprocation is not as visible and appreciable as the reciprocation of behavior, such as information seeking. Consequently, we tested the following hypothesis:

H8: The likelihood of formation of reciprocal information-seeking ties is beyond chance.

## Methods

### Design and setting

We used the data obtained in a cross-sectional network survey of a public health department in Ontario, Canada, serving a large urban population. The public health department had four organizational divisions dealing with specific public health issues, and one central supervisory/administrative division responsible for coordinating and overseeing the procedures. The practice-based divisions were chronic diseases, family health, environmental health, and communicable diseases. The department assigned ‘professional consultants’ to practice-based teams to conduct research, interpret and analyze data, and prepare specialized reports on the development and implementation of programs and policies. The survey was part of the baseline assessment of a study evaluating the impact of an organization-wide KT intervention on capacity for evidence-informed public health decision making [29].

### Sample

We invited all managers and professional consultants in the department to respond to an online survey through emails sent by the medical officer of health. Details of study methods and descriptive findings are provided elsewhere [30]. The managerial subgroup consisted of management staff, including the directors, medical officer of health, and managers. The professional consultant subgroup was composed of the staff who were specially trained to search for and appraise research evidence, and provide rapid evidence reviews to address practice-based problems. The staff in this subgroup consisted of the project specialists and epidemiologists.

### Data collection

We asked the respondents to identify the names of up to five staff in the health department to whom they regularly turned to get help applying research evidence to inform professional activities (information-seeking network); five staff who were experienced and knowledgeable in finding research evidence and translating it into practice (expertise-recognition network); and five staff whom they considered as personal friends (friendship network).

We defined the research evidence as ‘primary studies, systematic reviews, and meta-analyses that evaluate the effectiveness of an intervention’. In addition, we considered a broad range of activities fulfilling the criteria of ‘decision’; including decisions about how to implement programs/policies, how to address local issues that arise, and how to identify and respond to community needs.

### Analyses

We depicted the information-seeking and expertise-recognition networks with graphs in which the nodes represented the individuals and the directed lines (ties) between pairs of individuals showed whether the sender of the tie sought information from the receiver or recognized her as an expert. Basic indicators of structural composition of networks were calculated; which included the reciprocity and E-I index. Reciprocity is the tendency of network members to reciprocate the ties, and is presented as the fraction of all connections in the network that are reciprocal (bidirectional). E-I index is calculated for subgroups (partitions) of the network, and is the number of external connections minus the number of internal connections of the partition divided by all connections of that partition [31]. It ranges from–1 to 1. It captures the tendency of staff to bridge organizational boundaries and be connected to peers from other units.

We tested the effect of different personal and relational factors on the formation of information seeking and expertise-recognition ties between staff. Statistical analysis of social networks is more complicated than conventional statistical methods; because on the one hand, the assumption of independence of observations does not hold, and on the other hand, formation of ties between network members is under influence of various personal, dyadic, and broader structural and contextual factors [32]. A person may seek information from a peer because of her personal characteristics (*e.g.*, activity and socialization), the peer’s characteristics (such as expertise and prestige), their mutual attributes (*e.g.*, similarity in job and interests), and broader contextual factors (such as norms and common exposures). An appropriate statistical model should take these sources of variation into account. Decades of efforts in development of statistical techniques to address the sophisticated nature of social networks have resulted in the introduction of various models, each comes with its advantages and limitations [6]. Considering the sender and receiver effects as cross-nested random variables in a multi-level logistic regression to predict the formation of ties between the pair of actors (a.k.a. p2 model) is a partial solution to the dependence problem. It assumes dyadic independence but conditional on node level attributes (meaning that the formation of tie between two pairs of actors is independent, unless there is a common actor) [6]. A more advanced approach is exponential random graph model (ERGM) that uses the Markov chain Monte Carlo maximum likelihood method to estimate the network parameters based on more sophisticated dependence assumptions [6]. An important assumption of ERGM, which makes it distinct from conventional regression models, is the dependence assumption, postulating that the formation of a tie between any pair is also dependent on the existence of ties among other actors in the network. ERGM treats the ties (connections) between individuals as a random variable, and the observed pattern of ties as one realization of all possible random patterns. Consequently, the observed network is one realization of the set of all possible networks with similar size. Using the maximum likelihood (or associated) technique, it estimates the parameter values for the network configurations, in a way that maximizes the probability of the observed network structure. ERGM models are sophisticated, need specialized software, and are sometimes unstable [33].

In order to test the effect of personal and contextual factors in formation of information-seeking and expertise-recognition ties, we used two approaches; a multilevel logistic regression of dyadic associations, and an ERGM analysis.

The multilevel model was developed in STATA 12.1 program on a dataset in which the rows were all possible combinations of pairs of staff. The dependent variable was whether actor I seeks information from actor J or actor I identified actor J as an expert. The seeker and source identities were used as two cross-nested random variables; meaning that all seeker-source combinations exist in the dataset. Unlike a complete p2 model it did not include any network structural variables as predictors, and only predicted the likelihood that actor I sends a tie to actor J, ignoring the fact that actor I may be a source of information for J at the same time.

ERGM was carried out on the whole network matrices using SIENA program (version 3.11) that was joined with StOCNET graphical interface [34]. We developed two models to predict the formation of information seeking and expertise-recognition ties. As a measure of goodness of fit, a t-ratio was calculated for each independent variable in the model, indicating the difference between the observed pattern and the estimated value by the model. The t-ratio of less than 0.1 indicated good convergence [35]. The coefficients were compared to zero to assess if they significantly contributed in predicting the observed pattern.

### Variables and measures

We used three groups of variables in the stochastic analysis, as explained below:

### Node level variables

The variables about the characteristics of individuals were: being a manager (yes/no); being affiliated to the supervisory/administrative division (yes/no); and the affiliating organizational division (divisions 1 to 5). For each node level variable, we tested if the sender of the tie, the receiver of the tie, or both had that specific characteristic. For example, we tested the effect of being a manager and being affiliated to the supervisory/administrative division in predicting the information-seeking connections with the following variables:

1. ‘manager: seeker’; which tests if the managers were more likely to seek information, than professional consultants; and if managers were more likely to recognize others to be experts (testing hypothesis H1).

2. ‘manager: source’; which tests if the managers were more likely to be the source of information, than professional consultants; and if managers were more likely to be recognized as experts (testing hypothesis H1).

3. ‘manager: matching’; which tests the interaction effect of the tendency among managers to seek information from each other (testing hypothesis H5).

4. ‘Supervisory/administrative division: sender’; which tests if the staff of this division were more likely to seek information, than the staff of other divisions (testing for hypothesis H2).

5. ‘Supervisory/administrative division: receiver’; which tests if the staff of this division were more likely to be the source of information, than the staff of other divisions (testing for hypothesis H2).

6. ‘Supervisory/administrative division: matching’; which tests if the staff of this division were more likely to seek information from each other, than from other divisions (testing for hypothesis H4).

We measured the extent to which respondents implemented the principles of EBP in their daily activities using the EBP implementation scale, which was developed by Mazurek Melnyk *et al.* through a theoretically driven multi-stage process, and has been shown to have high internal consistency (a Cronbach’s alpha of 0.9) and test-retest reliability (0.94) on heterogeneous samples of nurses [36,37]. The implementation scale asked the respondents to provide the frequency of their involvement in 18 different EBP activities during eight weeks prior to the study, using a five-point frequency scale. The EBP activities included different aspects of using and appraising evidence to inform public health practice, and sharing the evidence with colleagues and clients. We tested the following three effects for the role of EBP implementation score:

1. ‘EBP score: seeker’; which tests if the staff with higher EBP implementation scores were more likely to seek information, than the staff with lower scores; and if the staff with higher scores were more likely to recognize others to be experts (testing for hypothesis H3).

2. ‘EBP score: source’; which tests if the staff with higher EBP implementation scores were more likely to be the source of information, than the staff with lower scores; and if the staff with higher scores were more likely to be recognized as experts (testing for hypothesis H3).

3. ‘EBP score: similarity’; which tests if there is a tendency in the staff with more similar EBP implementation scores to seek information from each other.

### Dyadic variables

We assessed the existence of friendship connections among the pairs of staff in the models, as an independent variable. For example, a positive parameter for friendship in the model predicting the information-seeking ties indicates that the information-seeking connections are more likely to happen among friends (testing hypothesis H6). We also tested the effect of being in the same division in formation of information seeking and expertise-recognition ties (testing for hypotheses H4).

### Structural parameters

For the endogenous characteristics of the social network, which shaped the dependence assumptions of the model, we included the reciprocity and the tendency of the network to contain some higher degree nodes (hubs) in the ERGM analysis.

### Reciprocity

Reciprocity is the tendency of person I to turn to person J, when J turns to I for seeking information. Positive reciprocity parameter in the model shows that there is a tendency towards formation of reciprocal (bi-directional) connections (testing hypothesis H8).

### Tendency for containing hubs

We assessed if there is a tendency in the network to contain a few nodes with significantly larger number of incoming or outgoing connections from others (hubs). To measure the tendency of some individuals to be the stars, the model should estimate the distribution of the nodes with the degrees (number of ties) of two, three, four, and so forth; which makes the model very complicated. Snijders *et al.* proposed alternating k-stars; which include high order stars (more than three ties), and are shown to improve the fitting of the model without overcomplicating it [38]. In a directed network, in which some individuals may either have large number of outgoing or incoming ties, these parameters are called alternating out-k-stars and alternating in-k-stars [33]. A positive coefficient for alternating out-k-stars parameter in predicting the information-seeking connections implies that there are a few highly active staff who seek information from many people. Similarly, a positive coefficient for alternating in-k-stars parameter suggests that, there are a few highly prestigious staff to whom several people turn to get information.

## Results

Out of 25 and 23 managers and professional consultants who participated in the survey, 15 and 13 answered all of the network questions, respectively (overall response rate of 58%). The characteristics of the study participants are shown in Table 1. Eighty-nine percent of participants were female. The managerial staff, in comparison to the professional consultants, had on average more than 10 years of work experience in public health. For two-thirds of managers, the highest attained degree was a Bachelors, while for approximately 70% of the professional consultants it was a Masters degree. On average, the professional consultants had higher scores on the EBP implementation scale.

**Table 1:** The baseline characteristics of study participants

Reciprocity (information seeking)	0.28	
Reciprocity (expertise recognition)	0.07	
E-I index (manager/prof)	0.15 (p:0.27)	
E-I index (divisions)	−0.45 (p < 0.0001)	
E-I index (supervisory/administrative division)	−0.45 (p = 0.22)	
	**Managers (n = 15)**	**Professional consultants (n = 13)**
Female (%)	14 (93)	11 (85)
Years of work experience in public health practice (SD)	20 (9)	10 (8)
Affiliated to supervisory/administrative division (%)	3 (20)	3 (23)
Highest degree		
baccalaureate	9 (60)	4 (31)
Masters and above	6 (40)	9 (69)
Evidence Based Practice implementation score (SD)	11 (5)	14 (8)
Outdegree (SD)	2 (1.3)	1.6 (1.2)
Indegree (SD)	1.5 (1.9)	2.2 (1.8)
Within group reciprocity	0.3	0.6
Between group reciprocity	0.3	0.5

**Figure 1:**
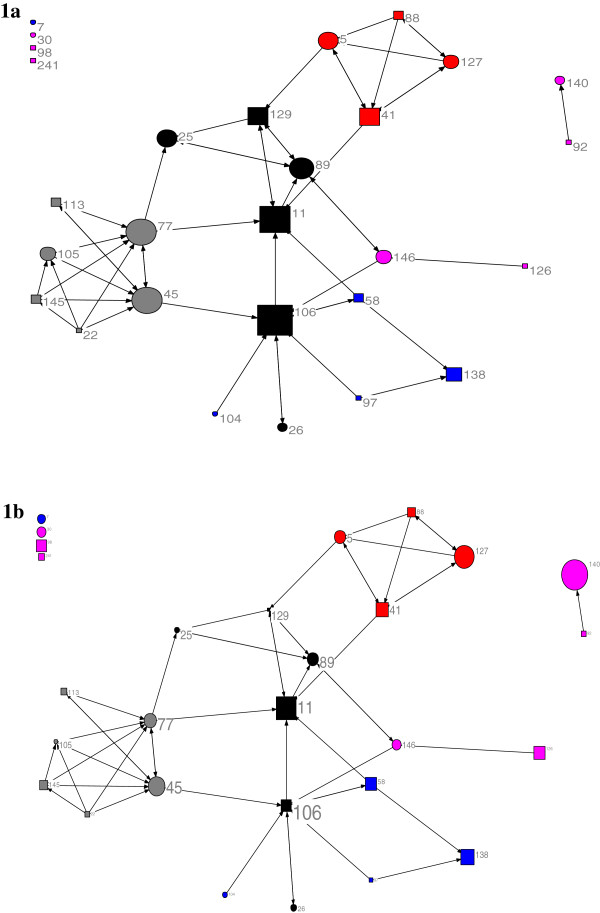
**The graph of the information-seeking network.** Circles: professional consultants; squares: managers; Colors indicate the divisions; Node size: **a**: indegree, **b**: the EBP implementation score.

Figure 1 shows the graph of the information-seeking network of these two groups (managers and professional consultants). As shown in Figure 1a, the staff of the supervisory/administrative division (black) were at the center of the map, with the highest centrality. Two managers in the supervisory/administrative division and two professional consultants in the grey division were the most central staff in the network. In addition, all interdivisional connections were limited exclusively to the information-seeking ties from divisions towards the supervisory/administrative division. Figure 1b, in which the size of the nodes is proportional to their EBP implementation scores did not show any visually recognizable pattern for the role of EBP implementation scores in the formation of information-seeking ties.

**Figure 2:**
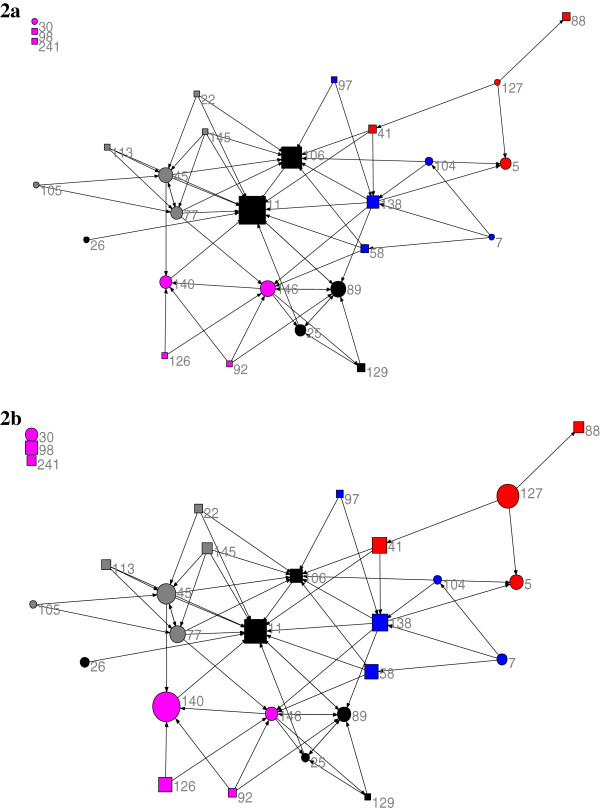
**The graph of the expertise-recognition network.** Circles: professional consultants; squares: managers; Colors indicate the divisions; Node size: **a**: indegree, **b**: the EBP implementation score.

Figure 2 shows the expertise-recognition network. Similar to the information-seeking network, two managers from the supervisory/administrative division were the most central staff in the network, and recognized as expert by the largest number of staff. These two managers were also the most central actors in the information-seeking map. According to Figure 2b, these managers had relatively high EBP implementation scores. However, there was not a visually recognizable pattern for the distribution of EBP implementation score in the expertise-recognition network. Unlike the information-seeking network, there were some inter-divisional connections, which did not include the supervisory/administrative division.

The reciprocity of information-seeking network (0.28) was higher than the expertise-recognition network (0.07), indicating that, the staff were more likely to reciprocate information seeking, but recognition of a peer as an expert was a mainly unidirectional connection. The E-I index of organizational divisions was significant and negative, meaning that the staff had a tendency to seek information from within their divisions, than turning to peers in other divisions.

We developed two models to predict the formation of information seeking and expertise-recognition ties using structural, node level, and dyadic variables. The coefficients for each parameter in the model represented the log odds ratio of the likelihood of formation of a tie conditional to one unit change in the independent variable. ERGM models showed a good convergence, with t-ratios of less than 0.1 for all parameters. The log odds ratios relevant to each independent variable and their statistical significance for multilevel regression and ERGM are shown in Tables 2 and 3 respectively.

**Table 2:** Multilevel logistic regression to predict information seeking and expertise-recognition ties, using node level, and dyadic variables

	**Information seeking coefficient (SE)**	**Expertise recognition coefficient (SE)**
Manager: seeker	0.66 (0.5)	−0.3 (0.4)
Manager: source	−0.3 (0.4)	−0.6 (0.6)
Manager: matching	−0.4 (0.4)	−0.2 (0.3)
Supervisory/admin division: seeker	−0.7 (0.7)	−2.0 (0.7)**
Supervisory/admin division: source	1.4 (0.6)*	2.9 (0.8)***
EBP score: seeker	−0.002 (0.04)	−0.01 (0.03)
EBP score: source	−0.01 (0.03)	0.2 (0.05)***
EBP score: absolute difference	0.007 (0.04)	−0.003 (0.03)
Division: matching	3.1 (0.5)***	2.5 (0.5)***
Expertise recognition	3.1 (0.5)***	-
friendship	2.4 (0.8)**	2.4 (0.8)**
Intercept	−5.5 (1.0)***	−6.4 (1.2)***
Random effect variance: seeker	0.43 (0.4)	0.3 (0.3)
Random effect variance: source	~0	1.4 (0.9)

Coefficients represent the log odds ratio (SE) of the likelihood of tie formation.

*: p < 0.05, **: p < 0.01, ***: p < 0.001.

**Table 3:** Exponential random graph model to predict information seeking and expertise-recognition ties, using structural, node level, and dyadic configurations

	**Information-seeking coefficient (SE)**	**Expertise-recognition coefficient (SE)**
Reciprocity	1.62 (0.7)*	−0.28 (0.8)
Alternating out-k-stars	0.22 (0.3)	0.51 (0.3)
Alternating in-k-stars	0.77 (0.4)*	1.44 (0.3)*
Manager: seeker	0.28 (0.4)	−0.12 (0.3)
Manager: source	−0.30 (0.3)	−0.06 (0.2)
Manager: matching	−0.009 (0.3)	0.18 (0.3)
Supervisory/admin division: seeker	−1.48 (0.6)*	−1.52 (0.6)*
Supervisory/admin division: source	1.65 (0.6)*	1.44 (0.4)*
EBP score: seeker	−0.01 (0.03)	0.01 (0.02)
EBP score: source	0.06 (0.03)*	0.08 (0.03)*
EBP score: similarity	0.90 (0.9)	1.53 (0.9)
Division: matching	2.96 (0.5)*	2.65 (0.5)*
Friendship	1.99 (0.7)*	2.18 (0.8)*

Coefficients represent the log odds ratio (SE) of the likelihood of tie formation.

*: p < 0.05.

### Managers and professional consultants

In the multilevel model (Table 2), the tendency of managers for seeking information (the variable manager: seeker) or being the source of information (manager: source) was not significantly different from professional consultants. ERGM (Table 3) showed similar findings. Similarly, there was not any significant difference between managers and professional consultants in terms of expertise recognition. Consequently, the analysis does not support hypothesis H1.

### Supervisory/administrative division

As shown in Table 2, multilevel regression showed a significant tendency in the staff of the supervisory/administrative division to be the source of information, as well as being recognized as experts. These findings were consistent with the ERGM analysis (Table 3). In addition, the negative parameter for the tendency of the staff of the supervisory/administrative division to turn to others for information or recognize them as expert (supervisory/administrative division: seeker) implies the existence of a hierarchical structure in the network, in which the staff of other divisions significantly tended to form unidirectional ties towards the staff of the supervisory/administrative division.

The significance of the coefficients for alternating in-k-stars in the ERGM analysis (Table 3) implies a tendency in some staff to take the role of hubs in the network. In other words, there were a few staff who were known as experts and information sources by many peers. From exploring the network graphs (Figures 1 and 2) we can speculate that most of these staff were in the supervisory/administrative division.

### EBP implementation

Multilevel regression showed that the staff were more likely to recognize peers with higher EBP implementation scores as experts (log odds = 0.2). ERGM analysis consistently showed a significant tendency in the staff to recognize peers with higher EBP scores as experts (log odds = 0.08), and also turn to them for seeking information (log odds = 0.06). ERGM analysis supported the hypotheses H3. The significant colinearity of the variable ‘expertise recognition’ and EBP scores can explain why EBP score was not a significant predictor of information seeking in the multilevel regression analysis.

### Small circles of accessible peers

The average outdegree (the number of ties towards others) for information-seeking and expertise-recognition networks were 2.0 and 1.6 respectively. Even though respondents had the opportunity to identify up to five peers, the maximum size of the lists was four (one respondent) in the information-seeking and five (one respondent) in expertise-recognition networks. In the ERGM analysis (Table 3), the coefficients for alternating out-k-stars was not statistically significant, suggesting that, there was not a significant dispersion in the number of information sources identified by the staff. Given the median of 2 for the number of information sources, we can suggest that, generally they preferred to limit their information sources to a couple of peers. Likewise, there was a tendency to limit the expertise-recognition ties to a few staff.

The positive parameter value for the matching of organizational divisions between the seeker and the source (division: matching) in both multilevel regression and ERGM analysis suggests that staff were more likely to seek information from peers in their own division. This measure was also statistically significant for expertise recognition, suggesting that, staff were more likely to recognize peers in their own division as experts. Consequently, the findings supported the hypothesis H4.

### Birds of a feather

The interaction effect for matching of job titles (manager: matching) tested whether managers formed professional ties within their own professional group. This term was not statistically significant in either network indicating that managers and consultants did not were just as likely to connect across roles as well as within role (H5 not supported). The findings did not support the existence of homophily of EBP practice either (hypothesis H7); meaning that the tendency of staff to turn to peers with similar EBP implementation scores was not significant.

The effect of friendship on the formation of information seeking and expertise-recognition ties was positive and statistically significant in both multilevel regression and ERGM analysis, supporting hypothesis H6. It either means that the staff tended to recognize someone as an expert if they were friends, or they tended to befriend experts.

Inclusion of both friendship and expertise recognition in predicting information seeking in the multilevel regression model let us study their adjusted effects. Both variables significantly predicted the formation of information-seeking ties (Table 2). Apart from the above-mentioned association between friendship and expertise recognition, it had an independent extra effect on the formation of information-seeking ties (log odds = 2.4). It implies that, some staff tended to seek information from their friends, even if they did not recognize them as experts. We could not include both friendship and expertise recognition in the ERGM analysis, because it resulted in degeneration of the model.

In the ERGM analysis, the information-seeking network also showed a statistically significant reciprocity, meaning that, staff tended to make their information-seeking connections bi-directional, supporting hypothesis H8. But, unlike the information-seeking network, the reciprocity was not statistically significant in the expertise-recognition network (if John recognized Bob as an expert, the likelihood that Bob also recognizes John as expert was not more than chance).

## Discussion

In this public health department, managers and professional consultants tended to seek information from a handful of peers who implemented EBP in practice, were their friends, and were in the supervisory/administrative division. In addition, the extent to which an individual implemented EBP in their practice significantly predicted whether others recognized them as an expert; along with being in the same organizational division, being in the supervisory/administrative division, and being friends.

The findings of multilevel regression and ERGM analysis were mostly consistent. ERGM analysis let us assess the effect of structural configurations on the formation of network ties. It showed a significant tendency in the staff to reciprocate the information-seeking ties, and also a tendency in some staff to serve as hubs in the network. Multilevel regression models let us assess the adjusted effect of friendship and expertise recognition on the formation of information-seeking ties. It showed an extra effect of friendship on information seeking, in addition to its association with expertise recognition.

### Organizational segregation

Our findings support the localized nature of interpersonal connections in the department, which means that the public health staff generally turn to a limited number of accessible peers to access information. This is reflected in the low density and clustered shape of the information-seeking and expertise-recognition networks. The tendency of health practitioners to limit their formal and informal networks geographically or professionally has also been confirmed by other studies [20,39,40]. This may be the result of the a tendency for health professionals to shape circles of trust by turning to easily accessible peers, with similar values and concerns, for getting information and discussing work-related issues (homophily) [21].

The relatively small networks of health practitioners and the structural segregation of information-seeking network is a possible barrier to the successful diffusion of organizational EIDM interventions. New knowledge and skills may get trapped in social clusters unless interdivisional communication is promoted, and more active participation of the staff from various divisions in educational interventions is encouraged.

### The significance of managers

Managers in our study did not tend to turn to professional consultants more than to other managers for information. This may imply that, in public health organizations, managers also frequently seek each other’s advice in order to implement research evidence into their decisions. The role of managers in provision of information about research evidence to other managers and some professional consultants underscores the pivotal role of managers as central staff of the divisions. In a study of hospital managers in acute-care hospitals in the United Kingdom, West and Barron found that managers had a considerable brokering role in connecting traditional clinical disciplines in their hospitals [20].

This suggests that involving managers in promoting EIDM in public health organizations is an important factor contributing to the success of KT interventions. Their significance is not only limited to administrative facilitators of organizational interventions. Their position as central actors in the information-seeking network makes them possible candidates for the early adoption of new KT knowledge and skills.

### Organizational readiness for evidence-informed practice

An important determinant of organizational change is the readiness of the organization for change [10]. French *et al.* in a comprehensive literature review developed a conceptual framework for the absorptive and receptive capacity of organizations [41]. Three major organizational attributes were vision, leadership, and a learning culture, and four stages of knowledge were need, acquisition of new knowledge, knowledge sharing, and knowledge use. Accordingly, in order to determine the readiness for change in an organization we need to assess how the staff acquire and share knowledge in daily practice as an indicator of organizational culture. In the current network analysis we went beyond assessing individual attitudes and self-perceived behavior by studying how the self-perceived behavior of the staff in implementing EBP is reflected in the behavior of their peers.

The findings suggest that the extent to which an individual implements the principles of EBP in practice is a significant predictor of being recognized as a potential source of information and expert by other peers. This finding implies that the self-perceived behavior of health practitioners is consistent with the judgment of peers; and the staff tend to seek information from peers who are known to be more evidence-informed. In other words, staff consider evidence-based behavior in choosing their sources of information and recognizing certain peers as experts. Consequently, the findings show a positive culture towards EIDM in the study health department, because the staff who implemented EBP in their practice were more likely to be chosen as experts and sources of information by others. This implies a generally positive attitude and practice towards EIDM in the managerial and professional staff, which is a potential facilitator of organizational change towards EIDM.

### Informal ties

Friendship was a significant predictor of information seeking. The importance of benevolence-based trust, which mainly happens within the context of friendship and intimacy, in formation of information-seeking ties has been shown in other studies [42]. People tend to limit their networks to their surrounding small circles to reduce the cost of obtaining information [21]. They prefer to seek information from the peers they know and trust well, even though they are not necessarily the most knowledgeable. The significance of friendship in predicting information seeking in the presence of expertise recognition in the regression model supported this hypothesis. An alternative explanation would be the role of awareness. The staff were more likely to seek information from friends, because they were more likely to be aware of their knowledge and expertise; while they did not have the opportunity to know about the capabilities of other peers.

### Implications

The findings of this study have specific implications for understanding the mechanisms of opinion leadership. The positive and significant association of friendship and expertise-recognition and information-seeking ties along with a localized pattern of social relations imply that there are staff in the health department who have the capability of influencing their peers through both formal and informal connections. These findings support the existence of local opinion leaders in the health department. Opinion leadership is the degree to which someone is able to informally affect others’ attitudes and behaviors in a desired way [43]. This position is not part of the formal role of people in an organization, but is earned by the person as a result of their competence, accessibility, trustworthiness, and conformity to social norms. Local opinion leaders hold strategic positions in the organization, and are suitable candidates to be chosen as early adopters of KT interventions. Flodgren *et al.* found that opinion leaders alone or in combination with other interventions may successfully promote evidence-informed practice, and the effect of opinion leaders is comparable with other well-known interventions such as distribution of educational materials and audit and feedback [44]. KT developers can take an active role and identify and convince the opinion leaders and use them as the agents of change in the organization.

The significance of friendship and similarity of workplace in predicting expertise-recognition ties implies that even for considering someone as an expert, which does not necessarily include any information exchange or communication with that person, the staff were affected by social proximity. This might be due to the fact that the respondents were more likely to be aware of someone’s expertise if they worked in a similar workplace and had more frequent interactions with them [15]. However, the significance of friendship in the presence of similarity of workplace in both regression and ERGM models suggests that strong social bonds (surrogated by friendship) are also important in recognizing a peer as expert.

We did not find any evidence of homophily by job title and EBP behavior. It could be explained by the fact that the participants of the study were only managers and professional consultants. At that level of decision making, the cost of playing social exchange games probably is not considerable or is worth paying.

### Limitations

This study is limited in different ways. Only 58% of the eligible staff participated in the survey, and non-respondents might have been different in terms of their interest in EIDM and their activity and prestige in social network. Low response rate might have also affected the quality of available data. Current picture of the network and the patterns of associations may not reflect the actual pattern in the health department, because not only the staff who did not respond but also their associations with each other and the participants are missed.

Causal inferences should also be interpreted with caution due to the cross-sectional nature of the study. The findings suggested that the staff who implemented EBP in their practice were more likely to be recognized as expert and be chosen as an information source. However, it is unclear that whether the implementation of EBP results in social recognition, or it is the necessities of social position that forces the central staff to improve their behavior towards EBP. A longitudinal study is needed to differentiate the effect of social selection (*i.e.*, being recognized by staff) from the context effect (*e.g.*, norms and job requirements).

In addition, limiting the maximum number of names each respondent could identify might have resulted in ceiling effect in the assessment of outdegrees; and the observed small outdegrees might be fictitious finding due to the ceiling effect.

## Conclusions

In summary, the findings showed that, the staff who implemented EBP in practice were more likely to be considered experts and information sources by their peers. The findings also suggest that, in public health organizations, the main factors contributing to the formation of information-seeking ties are both formal organizational structure (as reflected in the position of the supervisory/administrative division) and informal and tacit dynamics (as reflected in the effects of friendship and EBP scores). Similar factors contributed in recognizing a person as an expert. The network analysis provided clues to identify the organizational and social barriers and facilitators of change, organizational readiness for EIDM, and potential early adopters of KT interventions. The significance of friendship and workplace similarity in predicting expertise recognition implies that expertise recognition and accessibility are not cognitively distinct constructs, as suggested by current models of information seeking [14].

## Competing interests

The authors declare that they have no competing interests.

## Authors’ contributions

RYN and MD contributed to the design and conception of the study. RYN analyzed the data and drafted the manuscript. All authors contributed in interpretation of findings. MD and AM reviewed and suggested changes to the manuscript. All authors read and approved the final manuscript.
